# Age differences at sexual debut and subsequent reproductive health: Is there a link?

**DOI:** 10.1186/1742-4755-5-8

**Published:** 2008-10-31

**Authors:** Anu Manchikanti Gómez, Ilene S Speizer, Heidi Reynolds, Nancy Murray, Harry Beauvais

**Affiliations:** 1 Department of Maternal and Child Health, Gillings School of Global Public Health, University of North Carolina at Chapel Hill, CB 7599, Chapel Hill, NC, 27599-7599, USA; 2 Family Health International, Research Triangle Park, NC, USA; 3 Mathematica Policy Research, Inc, Washington, DC, USA; 4 Fondation pour la Sante Reproductrice et l'Education Familiale, Port-au-Prince, Haiti

## Abstract

**Background:**

Experiences at sexual debut may be linked to reproductive health later in life. Additionally, young women with older sexual partners may be at greater risk for HIV and sexually transmitted infections. This study examines sexual debut with an older partner and subsequent reproductive health outcomes among 599 sexually experienced women aged 15–24 who utilized voluntary counseling and testing or reproductive health services in Port-au-Prince, Haiti.

**Methods:**

Logistic regression models, controlling for socioeconomic and demographic factors, examined whether age differences at first sex were significantly associated with STI diagnosis in the previous 12 months and family planning method use at last intercourse.

**Results:**

Sixty-five percent of women reported sexual initiation with a partner younger or less than 5 years older, 28% with a partner 5 to 10 years older, and 7% with a partner 10 or more years older. There was a trend towards decreased likelihood of recent use of family planning methods in women who had first sexual intercourse with a partner 5 to 9 years older compared to women with partners who were younger or less than 5 years older. Age differences were not linked to recent STI diagnosis.

**Conclusion:**

Programs focusing on delaying sexual debut should consider age and gender-based power differentials between younger women and older men. Future research should examine whether wide age differences at sexual debut are predictive of continued involvement in cross-generational relationships and risky sexual behaviors and explore the mechanisms by which cross-generational first sex and subsequent reproductive health may be connected.

## Background

A woman's experience at sexual debut is associated with future reproductive health outcomes. Women who sexually debut at earlier ages are more likely to participate in high-risk behaviors and experience unintended pregnancy, HIV and sexually transmitted infections (STIs) [1-3]. Though research has examined how age differentials in current sexual relationships influence reproductive health, few studies have focused on how age differences at *first sexual intercourse *affect subsequent reproductive health outcomes.

Relationships between young women and older men are considered to be the norm in some cultural contexts. However, women in sexual relationships with older male partners have been found to have poor reproductive health outcomes, including increased risk for HIV infection [4-6]. Dissassortive sexual age mixing patterns can provide an entryway for HIV and STIs into the younger generation [6]. In the context of poverty and gender inequality, cross-generational sex often involves sex in exchange for money or goods (transactional sex), characterized by less condom use and greater sexual coercion [5,7-9].

The risks associated with cross-generational sex may be, in part, due to the power imbalances between the partners [10,11]. Particularly if a young woman is dependent on an older man for financial support, she may have little power to negotiate safe sex [8]. Furthermore, in instances where a young woman does assert herself, she may face sexual and physical violence [12,13].

One of the least developed countries in the world and the poorest in the Western hemisphere, Haiti has the highest HIV prevalence in Latin America and the Caribbean. HIV prevalence for females was estimated to be 2.3% in 2005–06 with the highest rates among women aged 25–29 (3.5%) and 30–35 (4.1%) [14]. The HIV epidemic in Haiti is fueled by an unstable economy and violence against women: in 2000, 16% of women living in union reported experiencing sexual violence in the past 12 months [15]. Economic insecurity forces women to initiate sexual activity earlier and to depend on sexual relationships for financial support, often with older men [16,17]. To date, little is known about the circumstances and ramifications of power imbalances and transactional sex experienced by young women in Haiti.

While it is apparent that partner age differences in current or recent relationships may affect women's reproductive health, few studies have explored the effect of partner's age at sexual debut on subsequent reproductive health outcomes. This study begins to fill this gap by examining the association between cross-generational first sex and reproductive health outcomes among women aged 15–24 in Port-au-Prince, Haiti.

## Methods

Data for this study were collected in 2004 from youth using one of the five Fondation pour la Sante Reproductrice et l'Education Familiale (FOSREF) facilities in Port-au-Prince, Haiti. Women and men aged between 15–24 years visiting one of four youth centers for reproductive health services, voluntary counseling and HIV testing (VCT) or to receive condoms from the facility-based distribution site were approached for interview after they had received services or obtained condoms. Additionally, young women visiting a reproductive health clinic serving women of all ages were approached for interview after receiving services. Prior to initiating the interview, interviewers read a consent statement and received written informed consent from all respondents. The exit interviews were conducted in Haitian Creole language by trained interviewers from FOSREF using a structured questionnaire. The methods of the study have been described in further detail elsewhere [18]. A total of 478 young men and 807 young women were interviewed. This secondary data analysis utilizes a subset of 599 young women who had initiated sexual activity at the time of the interview. Women were excluded if they did not report initiating sexual intercourse (n = 84) and if information about her age and/or her partner's age at first sexual intercourse was missing (n = 144).

The independent variable of interest is categorical and describes cross-generational first sex, or the difference between the age of the respondent and her first sexual partner. Respondents were asked, "What was the age of the person with whom you had your first sexual intercourse?" Responses were categorized into three binary variables: younger than the woman or less than 5 years older, 5–9 years older, and 10 or more years older. While there are no standard age difference categories for studying cross-generational sex [8], these age groupings were formulated based on other studies examining sexual mixing patterns between older men and younger women [4,5,7].

Outcomes of interest in our study were binary variables indicating whether the respondent reported being diagnosed with an STI in the previous 12 months and use of family planning, including modern and traditional methods, at last sexual intercourse. The following sociodemographic characteristics were included as categorical variables in all multivariate logistic regression models: number of years since sexual initiation (0–2 years, 3–5 years, and 6 or more years); religion (Catholic, Protestant, and other); current age (15–18, 19–22, and 23–24); employment status; education level (primary, secondary, and higher education completed); and type of facility used (youth center or reproductive health clinic for all ages). Additionally, a dummy variable indicated whether the respondent was in union (married, living with her partner, or *placé*, a socially binding union involving economic and sexual exchanges or a common-law marriage in Haiti) [19]. Principal components analysis was used to create a standards of living index as a proxy measure for socioeconomic status for the entire sample of men and women [20]. Low socioeconomic status was defined as being in the lowest 40% of the index, medium in the next 40%, and high for the top 20%.

Descriptive statistics were calculated. Cross-tabulations of age differences and circumstances of sexual initiation were used to provide greater understanding of the first sexual encounter. Pearson's chi square tests were used to assess significant differences in sexual initiation by partner age difference. Bivariate and multivariate logistic regression models were used to examine the relationship between cross-generational first sex and reproductive health outcomes. All analyses were performed using STATA version 9.2.

## Results

Among the 599 women included in this study, the mean age was 20.5 years. Twenty-eight percent experienced first sexual intercourse with a partner 5 to 9 years older, and 7% with a partner 10 or more years older (Table 1). The majority of respondents (65%) initiated sexual activity with a partner younger or fewer than 5 years older. Less than 13% reported being diagnosed with an STI in the previous year, and 42% used a family planning method at last sexual intercourse. More than half of the participants had been pregnant before, and 56% reported living in union. The mean age at first sexual intercourse was 16.3 years, and less than 20% of respondents initiated sexual activity before the age of 15. Most women (76%) had completed secondary education.

**Table 1:** Characteristics of sexually experienced, female VCT and reproductive health clients aged 15–24, Port-au-Prince, Haiti, 2004

**Age difference between respondent and first sexual partner**	% (n)
≤ 4 years	65.3 (391)
5 years to 9 years	27.6 (165)
≥ 10 years	7.2 (43)

Diagnosed with an STI in past 12 months*	12.5 (75)

**Used a family planning method at last sex**	42.2 (253)

**Has ever been pregnant**	56.9 (341)

**Clinic type**	

Women's reproductive health	24.9 (149)
Youth center	75.1 (450)

**Age of sexual initiation**	

≤ 14	19.5(117)
15–17	52.4 (314)
≥ 18	28.1 (168)

**Years since sexual initiation**	

0–2	29.2 (175)
3–5	39.6 (237)
≥ 6	31.2 (187)

**Socioeconomic status**	

Highest	18.4 (110)
Middle	35.6 (213)
Lowest	46.1 (276)

Religion*	

Catholic	51.6 (309)
Protestant	33.6 (201)
Other	13.5 (81)

Employed*	18.5 (111)

**Current age**	

15–18	22.9 (137)
19–22	50.1 (300)
23–24	55.9 (162)

Relationship status*	

Not in union	42.7 (256)
In union	55.9 (335)

Highest level of education completed*	

Primary	13.2 (79)
Secondary	76.3 (457)
Higher	7.4 (44)

Notes: n = 599. *The following variables were missing data (n in parentheses): STI (81); religion (8); employment status (2); relationship status (8); and education (19).

A higher proportion of women with partners 5 to 9 years older initiated sex before the age of 15, as compared to women with partners less than 5 years older or 10 or more years older (Table 2). Women who had their first sexual experience with a partner 10 or more years older seemed to have sexually debuted at older ages than women with younger partners; these partners would have been close to their thirties or older. These differences were not statistically significant. Among women with first sexual partners 10 or more years older, 5% described their partner as their husband, while less than 1% of women with partners less than 5 years older and 2% with partners 5 to 9 years older did so. Among respondents with partners less than 5 years older, 96% described the first partner as a boyfriend, compared to 95% of women with partners 5 to 9 years older and 88% with partners 10 or more years older. These differences were marginally significant (p = .091). A slightly higher proportion of women who were closer in age to their first sexual partners (<5 years age difference) used a condom at first sex (23%) as compared to women with older partners. These differences were not statistically significant.

**Table 2:** Characteristics of sexual initiation by first sexual partner's age

Age difference between respondent and partner at first sex	Younger or less than 5 years older	5 to 9 years older	10 or more years older
	% (n)	% (n)	% (n)
**Age of sexual initiation**			

≤ 14	18.7 (73)	23.0 (38)	14.0 (6)
15–17	54.2 (212)	49.7 (82)	46.5 (20)
≥ 18	27.1 (106)	27.3 (45)	39.5 (17)
*χ^2 ^p-value*	*0.307*		
*Mean (yrs)*	16.3	16.2	16.9

**Age difference between respondent and partner**			

*Mean (yrs)*	2.1	6.3	13.0

Relationship to first sexual partner*			

Husband	0.5 (2)	1.8 (3)	4.9 (2)
Boyfriend	96.4 (376)	95.2 (157)	87.8 (36)
Friend	2.3 (9)	1.2 (2)	4.9 (2)
Other	0.8 (3)	1.8 (3)	2.4 (1)
*χ^2 ^p-value*	*0.091*		

**Used a condom at first sex**			

Yes	23.0 (90)	17.6 (29)	18.6 (8)
No	77.0 (301)	82.4 (136)	81.4 (35)
*χ^2 ^p-value*	*0.326*		

Notes: n = 599. *3 women were missing data.

In bivariate logistic regression models, women who reported having a partner 5 to 9 years older at first sex were less likely (OR: 0.67, 95% CI: 0.46–0.98) to report using a family planning method at last sex, compared to women whose first sexual partner was younger or less than 5 years older (Table 3). Having a partner 10 or more years older did not have a statistically significant effect on family planning use. There was no significant relationship between age differences at first sex and recent STI diagnosis in the bivariate model.

**Table 3:** Odds ratios (and 95% confidence intervals) from bivariate logistic regression models assessing the association of reproductive health outcomes with cross-generational first sex

	STI diagnosis in previous 12 months n = 518	Did not use a family planning method at last sex n = 590
**Age difference between respondent and first sexual partner**		

≤ 4 years	1.00	1.00
5 years to 9 years	1.00 (0.57 – 1.76)	0.67 (0.46 – 0.98)*
≥ 10 years	1.65 (0.71 – 3.81)	0.93 (0.49 – 1.78)

Notes: 81 respondents were missing data on STI diagnosis, and 9 were missing data on family planning method use at last sex. * p < 0.05.

Controlling for sociodemographic and reproductive characteristics, women who sexually debuted with partners 5 to 9 years older were less likely to report using a family planning methods at last sex (OR: 0.67, 95% CI: 0.44 – 1.00; Table 4). This relationship was not statistically significant (p = 0.052). Other factors associated with non-use of a family planning method at last sexual intercourse included pregnancy experience and being Catholic, while female youth who were employed were significantly more likely to report using family planning. Having a partner 10 or more years older did not have a significant influence on family planning use, nor did age differences have an impact on recent STI diagnosis.

**Table 4:** Odds ratios (and 95% confidence intervals) from multivariate logistic regression models assessing the association of reproductive health outcomes with cross-generational first sex

	STI diagnosis in previous 12 months n = 489	Did not use a family planning method at last sex n = 558
**Age difference between respondent and first sexual partner**		

≤ 4 years	1.00	1.00
5 years to 9 years	0.93 (0.51 – 1.69)	0.67 (0.44 – 1.00)†
≥ 10 years	1.49 (0.61 – 3.66)	1.05 (0.52 – 2.10)

**Years since sexual debut**		

≥ 6 years	1.00	1.00
3–5 years	0.79 (0.40 – 1.55)	1.08 (0.67 – 1.74)
1–2 years	0.74 (0.33 – 1.66)	1.10 (0.63 – 1.93)

**Ever pregnant**		

No	1.00	1.00
Yes	1.28 (0.69 – 2.36)	0.49 (0.32 – 0.75)***

**Clinic type**		

Youth center	1.00	1.00
Women's reproductive health	0.40 (0.18 – 0.86)*	1.18 (0.75 – 1.87)

**Socioeconomic status**		

Highest	1.00	1.00
Middle	0.84 (0.41 – 1.68)	0.87 (0.52 – 1.45)
Lowest	0.59 (0.27 – 1.26)	0.75 (0.44 – 1.28)

**Religion**		

Catholic	1.00	1.00
Protestant	0.84 (0.46 – 1.54)	0.72 (0.48 – 1.06)
Other	1.55 (0.73 – 3.31)	0.46 (0.26 – 0.80)**

**Employment status**		

Not employed	1.00	1.00
Employed	0.62 (0.28 – 1.36)	2.05 (1.23 – 3.42)**

**Current age**		

15–18	1.00	1.00
19–22	1.48 (0.69 – 3.20)	0.95 (0.59 – 1.54)
23–24	1.52 (0.59 – 3.93)	0.91 (0.49 – 1.69)

**Relationship status**		

Not in union	1.00	1.00
In union	1.62 (0.90 – 2.92)	0.88 (0.59 – 1.31)

**Education level**		

Primary	1.00	1.00
Secondary	1.41 (0.53 – 3.73)	0.83 (0.47 – 1.46)
Higher	1.35(0.36 – 5.09)	1.20 (0.49 – 2.91)

Notes: N's vary from Tables 3 due to missing data on control variables.

†p = 0.052. * p < 0.05. ** p < 0.01. *** p < 0.001

## Discussion

This is one of the few studies to examine partner age differences at sexual debut and subsequent reproductive health outcomes in a developing country. Most studies focus on age differences between young women and current partners [21,22]. While examining an understudied topic in a unique population, this analysis was limited by the cross-sectional, non-representative nature of the sample. We were unable to establish a causal relationship between age differences at first sex and later reproductive health behaviors. Respondents were not surveyed about other key risk behaviors, such as the lifetime number of sexual partners or age of current sexual partners. Family planning method use at last sexual intercourse captures one event in the respondent's sexual history and is not necessarily representative of their experiences. Additionally, 17% of sexually experienced women were excluded from this analysis due to missing data on age of sexual initiation and/or age of first sexual partner. Though most women with missing data reported their first sexual partner to be a boyfriend, perhaps their lack of knowledge about this partner's age may reflect a more casual and potentially more risky relationship.

The few studies that have examined cross-generational first sex and its relation to subsequent reproductive health have shown mixed results. Examining a longitudinal, representative sample of adolescents in the United States, Ryan and colleagues found that women who initiated sex before the age of 16 with an older partner had a greater likelihood of testing positive for an STI in young adulthood. Early, cross-generational first sex did not have a statistically significant impact on teenage or non-marital birth [21]. Another U.S. study, using a non-representative sample of adolescents, found that women who initiated sex with an older partner were significantly more likely to have been pregnant during their lifetimes and less likely to use condoms at first sex, last sex and during their lifetimes, although there was no difference in STI diagnosis or use of birth control methods [22]. Varying definitions of what constitutes an older partner limits this body of research, and future work should utilize representative data in settings beyond the United States to better establish whether age differences at first sex have health effects later in life.

While our study is unable to examine causal mechanisms that may connect sexual initiation and reproductive health later in life, we hypothesize that the lower likelihood of family planning method use at last sex among women whose first sexual partner was 5 to 9 years older may be potentially due to an imbalance in power differentials that began at sexual debut. Previous research suggests that sexual experiences with an older partner may be coercive and/or disempowering [13,23], possibly setting a relationship pattern which limits a woman's ability to negotiate for condom and contraceptive use later in life [10]. Power imbalances may lead to a lack of communication between partners, thus limiting a young woman's ability to advocate for condom and contraceptive use [10]. While the data utilized in this study did not have any measures of gender-based power in current or recent relationships, future research in this area should consider power as a mediator between cross-generational first sex and subsequent reproductive health outcomes. Additionally, since forced sex is not uncommon in Haiti [15,24,25], exploring whether cross-generational sex involves coercion could further explain the circumstances of sexual experiences and how they may influence later reproductive health.

## Conclusion

Though this study does not provide strong support to the hypothesis that cross-generational first sex influences later reproductive health, future research should utilize representative samples of youth in developing countries and examine the context of first sex, as well as gender-based power as a pathway to adverse reproductive health. The influence of risky circumstances at sexual debut, specifically age differentials between partners, on sexual risk taking behavior later in life should be explored, investigating also the motivations of younger women to engage in relationships with older men. Additionally, research should investigate whether age differences between partners at first sex are associated with cross-generational sex later in life. HIV and teen pregnancy prevention programs in Haiti and elsewhere where premarital sex is common should focus on delaying sexual debut but also consider age and gender-based power imbalances, particularly among economically vulnerable young women.

## Competing interests

The authors declare that they have no competing interests.

## Authors' contributions

AMG conceived and designed this research paper, conducted data analysis, and drafted and revised the manuscript. ISS and HR participated in designing the study, interpretation of results, and drafting and critically reviewing the manuscript. NM, at the time of paper writing, was based at the lead organization that initiated the research study. She designed the study and reviewed the manuscript. HB is from the local implementing firm and played a key role in collecting the data and revising the manuscript. All authors read and approved the final manuscript.
